# DOK3 maintains intestinal homeostasis by suppressing JAK2/STAT3 signaling and S100a8/9 production in neutrophils

**DOI:** 10.1038/s41419-021-04357-5

**Published:** 2021-11-06

**Authors:** Jia Tong Loh, Koon-Guan Lee, Alison P. Lee, Joey Kay Hui Teo, Hsueh Lee Lim, Susana Soo-Yeon Kim, Andy Hee-Meng Tan, Kong-Peng Lam

**Affiliations:** 1 Singapore Immunology Network, Agency for Science, Technology and Research, 8A Biomedical Grove, Singapore, 138648 Republic of Singapore; 2 Bioprocessing Technology Institute, Agency for Science, Technology and Research, 20 Biopolis Way, Singapore, 138668 Republic of Singapore; 3 Department of Microbiology and Immunology, Yong Loo Lin School of Medicine, National University of Singapore, 5 Science Drive 2, Singapore, 117545 Republic of Singapore; 4 School of Biological Sciences, College of Science, Nanyang Technological University, 60 Nanyang Drive, Singapore, 637551 Republic of Singapore

**Keywords:** Neutrophils, Mucosal immunology

## Abstract

How pathogenesis of inflammatory bowel disease (IBD) depends on the complex interplay of host genetics, microbiome and the immune system is not fully understood. Here, we showed that Downstream of Kinase 3 (DOK3), an adapter protein involved in immune signaling, confers protection of mice from dextran sodium sulfate (DSS)-induced colitis. DOK3-deficiency promotes gut microbial dysbiosis and enhanced colitis susceptibility, which can be reversed by the transfer of normal microbiota from wild-type mice. Mechanistically, DOK3 exerts its protective effect by suppressing JAK2/STAT3 signaling in colonic neutrophils to limit their S100a8/9 production, thereby maintaining gut microbial ecology and colon homeostasis. Hence, our findings reveal that the immune system and microbiome function in a feed-forward manner, whereby DOK3 maintains colonic neutrophils in a quiescent state to establish a gut microbiome essential for intestinal homeostasis and protection from IBD.

## Introduction

Inflammatory bowel disease (IBD) is a chronic immune-mediated inflammatory disorder of the gastrointestinal tract characterized by periods of remission and relapse. IBD constitutes a significant health burden in developed countries since the twentieth century, and their incidence is rising in developing countries. Recent genome-wide association studies (GWAS) have identified 163 host genetic loci which show significant association with IBD, and these genes can be classified into several key pathways including innate immunity (e.g., *NOD2*), cytokine signaling (e.g., *JAK2*, *STAT3*, and *TYK2*), and the maintenance of intestinal barrier integrity (e.g., *HNF4A*, *CDH1*, and *MUC19*) [1, 2]. Nevertheless, such susceptibility loci could only account for approximately 20% of IBD risk, suggesting that apart from host genetic factors, the etiology of IBD is also strongly influenced by the gut microenvironment, including the composition of the gut microbiome [3].

The intestine is colonized by a wide spectrum of bacteria, fungi and protozoa, and the composition of microbiota is a strong determinant of gut health and disease. Commensal microorganisms help to shape our gut immune system, and actively protects against other pathogenic microbes through colonization resistance and synthesis of factors which promote mutualism [4–6]. As such, microbial dysbiosis, resulting from an imbalance between pathogenic and beneficial bacteria, is associated with intestinal inflammation and diseases such as IBD and colorectal cancer [7]. Indeed, clinical studies revealed that the global gut microbiota composition, in particular microbial diversity and relative abundance of specific bacterial taxa, is vastly different between IBD patients and healthy individuals, indicating a strong association between gut microbiome and disease.

Intestinal homeostasis depends critically in part on the crosstalk between gut microbiota and the innate immune system, and a dysregulated immune response has frequently been shown to be a key driver of IBD [8, 9]. Innate immune cells such as dendritic cells (DCs), macrophages and neutrophils express a variety of pattern recognition receptors (PRRs) such as Toll-like receptors (TLRs) and C-type lectin receptors (CLRs) for the sensing of microbial-associated molecular patterns and/or host-derived damage-associated signals. Upon sensing of microbiota in the gut, multiple signaling pathways including those of NF-κB, MAPK, and inflammasome will be activated, leading to a range of protective or inflammatory immune responses such as cytokine production and the polarization of immune cell subsets. Thus, the interaction between the innate immune system and gut microbiota is critical for the maintenance of intestinal homeostasis and protection against IBD. However, the precise mechanism by which the immune system shapes the gut microbial community remains largely unknown.

Downstream of Kinase 3 (DOK3) is an adapter molecule which limits tyrosine kinase-mediated signaling downstream of various immuno-receptors [10]. Due to their lack of intrinsic enzymatic activity, they function predominantly as a molecular scaffold to nucleate protein complexes during signal transduction. DOK3 is highly expressed in certain immune cells, and accumulating evidence demonstrates its functional importance downstream of numerous PRRs during innate immune responses. DOK3 suppresses Card9 signaling downstream of CLR during fungal infection in neutrophils [11], while they negatively regulate lipopolysaccharide (LPS) sensing through the TLR4-ERK axis in macrophages [12]. Recently, GWAS studies revealed that *Dok3* gene resides within a susceptibility gene region for Crohn’s disease and ulcerative colitis that also harbors many other immune-related genes [1]. However, a causal relationship between DOK3 and IBD remains unclear.

In this study, we examined the physiological role of DOK3 in IBD. We found that *Dok3*-knockout (*Dok3*^*−/−*^) mice are highly susceptible to DSS-induced colitis. Mechanistically, we demonstrated that DOK3 restrains JAK2/STAT3 signaling in colonic neutrophils, thereby limiting S100a8/9 expression, to establish a protective gut commensal microbial community. Together, our results revealed a previously unappreciated role of DOK3 in enforcing a quiescent state in colonic neutrophils, which modulates gut microbiota and promotes intestinal homeostasis.

## Results

### Loss of DOK3 exacerbates colitis

**Fig. 1:**
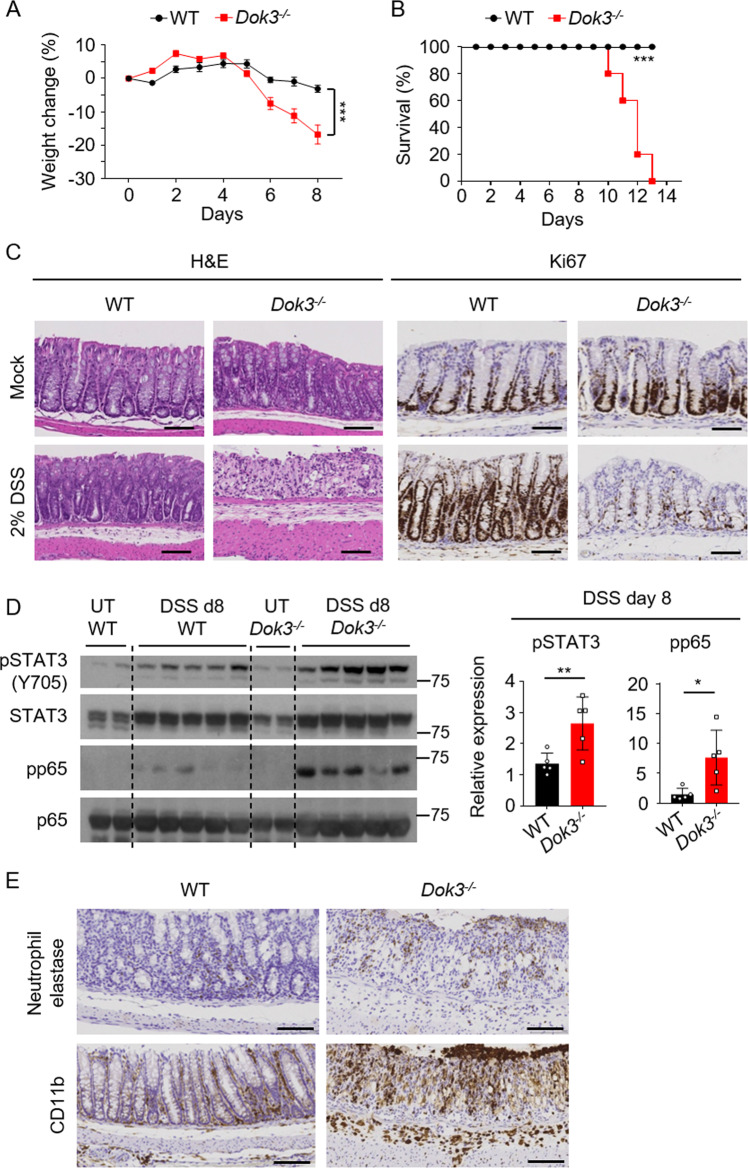
Loss of DOK3 enhances susceptibility to DSS-induced colitis. WT and *Dok3*^*−/−*^ mice were given 2% DSS in their drinking water for 7 days. **A** Body weight and **B** survival were scored daily. **A** Data is shown as mean±S.E.M (*n* = 5). ***p < 0.0001, two-way repeated-measures ANOVA. **B** ***p = 0.0002, log-rank test. **C** Colon tissues were collected on day 8 and stained with H&E or Ki67. Scale bars, 100 µm. **D** Immunoblot analysis (left) and densitometry (right) of phosphorylated and total STAT3 and NF-κB p65 in the colons of untreated (UT) or DSS-treated mice (day 8). Each lane in the immunoblot represents one sample examined. Data is shown as mean ± SD (*n* = 5). *p = 0.02, **p = 0.01, unpaired two-tailed Student’s *t*-test. **E** Colon tissues were collected on day 8 and stained with neutrophil elastase or CD11b. Scale bars, 100 µm.

A previous GWAS study of Crohn’s disease and ulcerative colitis implicated a possible role for DOK3 in IBD by virtue of its localization in a susceptibility gene region that also harbors a few other immune-related genes [1]. Hence, to determine if a causal relationship between DOK3 and IBD exists, we induced experimental colitis in wild-type C57BL/6 (WT) and *Dok3*^−/−^ mice via oral administration of 2% DSS. Conventionally raised *Dok3*^−/−^ mice displayed enhanced susceptibility to colitis as evidenced from their greater weight loss and reduced survival compared with WT mice (Fig. 1A, B). *Dok3*^−/−^ mice also exhibited shorter colon lengths, bloody diarrhea, and rectal bleeding (Supplementary Fig. 1A, B). Histological analyses revealed complete loss of crypts and ulceration in the colons of *Dok3*^−/−^ mice following DSS treatment (Fig. 1C, left). In addition, their mucosal barrier was depleted, resulting in increased bacterial penetration into the mucous layer (Supplementary Fig. 1C). Colonic sections from *Dok3*^−/−^ mice also stained for a lower level of the proliferative marker Ki67 (Fig. 1C, right), indicative of impaired tissue regeneration in the absence of DOK3. Moreover, enhanced activation of STAT3 and NF-κB (Fig. 1D), as well as increased infiltration of neutrophils (visualized via staining for neutrophil elastase) and CD11b^+^ myeloid cells (Fig. 1E), were observed in the colons of *Dok3*^−/−^ mice. Together, these data show that DOK3 protects against DSS-induced acute colitis by limiting colonic inflammation.

### DOK3 deficiency promotes a dysbiotic microbiome

**Fig. 2:**
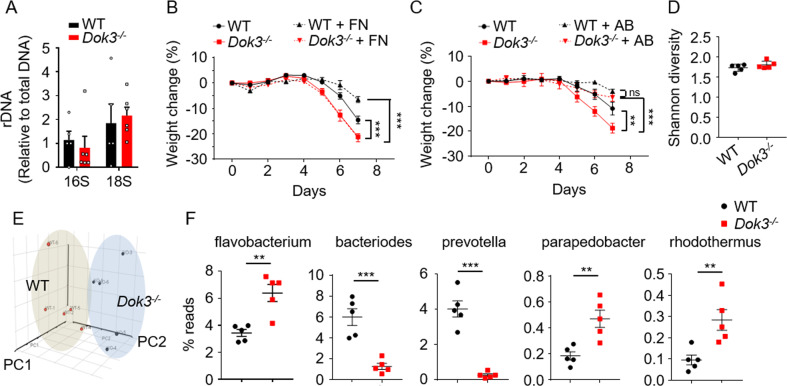
DOK3 deficiency results in a dysbiotic colonic bacterial microbiome. **A** RT-qPCR analysis of 16S and 18S rDNA in fecal DNA obtained from WT and *Dok3*^*−/−*^ mice. Data is shown as mean ± SEM (*n* = 5–6). **B** WT and *Dok3*^*−/−*^ mice were given fluconazole (FN) in their drinking water for 2 weeks prior to the induction of DSS colitis. Body weights were measured daily. Data is shown as mean ± SEM (*n* = 5). ***p < 0.0001, two-way repeated-measures ANOVA. **C** WT and *Dok3*^*−/−*^ mice were given antibiotics (AB) cocktail (vancomycin, ampicillin, neomycin, and metronidazole) in their drinking water for 4 weeks prior to the induction of DSS colitis. Body weights were measured daily. Data is shown as mean ± SEM (*n* = 5). **p = 0.01, ***p = 0.0005, two-way repeated-measures ANOVA. **D**–**F** High throughput sequencing of 16S rRNA in fecal bacterial DNA. **D** Shannon diversity of WT and *Dok3*^*−/−*^ mice fecal microbiota. Data is shown as mean ± SD (*n* = 5). **E** Principal-component analysis of the microbiota composition in WT and *Dok3*^*−/−*^ mice. Each symbol represents an individual mouse. **F** Relative abundance of major bacterial genera in WT and *Dok3*^*−/−*^ mice fecal microbiota. Data is shown as mean ± SEM (*n* = 5). **p = 0.002, 0.004, 0.008, ***p = 0.0005, <0.0001 (from left to right), unpaired two-tailed Student’s *t*-test.

Mounting evidence suggests that the pathogenesis of IBD is associated with a dysbiotic intestinal bacterial and fungal microbiota [13–15]. To understand if DOK3 deficiency affects the microbial landscape in the gut, we first measured and found no significant difference between the fecal bacterial and fungal load in the gut of WT and *Dok3*^*−/−*^ mice as assessed by 16S and 18S rDNA amplification, respectively (Fig. 2A). To decipher the contribution of bacterial and fungal microbiota to the enhanced susceptibility of *Dok3*^*−/−*^ mice to colitis, we fed WT and *Dok3*^*−/−*^ mice ad libitum with either a broad-spectrum antibiotics cocktail (consisting of vancomycin, ampicillin, neomycin, and metronidazole) or antifungal fluconazole to suppress the growth of bacteria or fungi species in their gut, respectively. *Dok3*^*−/−*^ mice treated with fluconazole showed comparable disease susceptibility as untreated *Dok3*^*−/−*^ mice upon colitis induction, suggesting that gut fungi do not contribute to increased colonic inflammation in *Dok3*^*−/−*^ mice during colitis (Fig. 2B). On the other hand, treatment with antibiotics is able to completely rescue the colitis susceptibility of *Dok3*^*−/−*^ mice, suggesting that altered gut microbiota is responsible for the exacerbated colitis in *Dok3*^*−/−*^ mice (Fig. 2C). As such, we performed 16S rRNA sequencing of fecal bacteria genomic DNA isolated from untreated WT and *Dok3*^*−/−*^ mice to characterize their bacterial microbiota. Even though the alpha diversities of WT and *Dok3*^*−/−*^ microbiota were comparable (Fig. 2D), community compositions significantly differed (Fig. 2E). Higher abundance of the genera flavobacterium, parapedobacter and rhodothermus, and lower abundance of bacteriodes and prevotella, were observed in *Dok3*^*−/−*^ mice as compared to WT mice (Fig. 2F). Notably, such microbiome profile was found largely to be similar to those seen in IBD patients [16–18]. Thus, loss of DOK3 promotes a dysbiotic, and possibly, more colitogenic bacterial microbiota in the gut.

### Microbiome regulates colitis susceptibility in *Dok3*^*−/−*^ mice

**Fig. 3:**
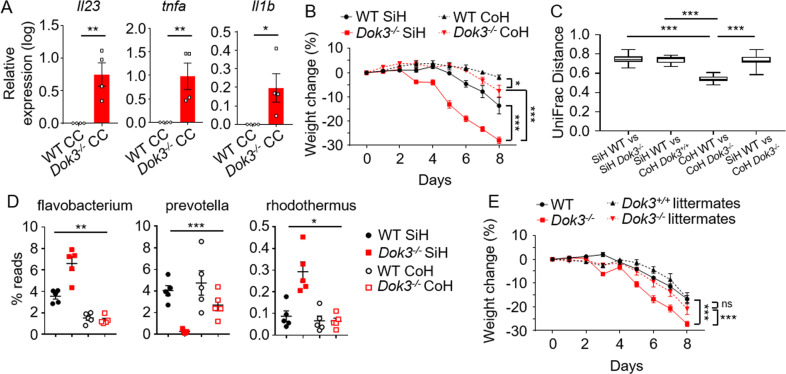
Microbiome in *Dok3*^*−/−*^ mice regulates colitis susceptibility. **A** RT-qPCR analysis of *Il23*, *tnfa*, and *il1b* expression relative to *b-actin* expression in WT lamina propria cells following 3 h stimulation with cecal contents (CC) from WT or *Dok3*^*−/−*^ mice. Data is shown as mean ± SEM (*n* = 4). *p = 0.04, **p = 0.005, 0.01 (from left to right), unpaired two-tailed Student’s *t*-test. **B** Singly-housed (SiH) or co-housed (CoH) WT and *Dok3*^*−/−*^ mice were given 2% DSS in their drinking water for 7 days and their body weights measured daily. Data is shown as mean ± SEM (*n* = 5). *p = 0.03, ***p < 0.0001, two-way repeated-measures ANOVA. **C** Unweighted UniFrac distance between SiH and CoH WT and *Dok3*^*−/−*^ mice (*n* = 5). ***p < 0.0001, Kruskal–Wallis test with Dunn’s post-hoc test. **D** Relative abundance of major bacterial genera in singly-housed (SiH) or co-housed (CoH) WT and *Dok3*^*−/−*^ mice fecal microbiota. Data is shown as mean ± SEM (*n* = 5). *p = 0.02, **p = 0.006, ***p = 0.0001, two-way ANOVA. **E** *Dok3*^*+/+*^ and *Dok3*^*−/−*^ littermates or non-littermates were given 2% DSS in their drinking water for 7 days and their body weights measured daily. Data is shown as mean ± SEM (*n* = 5). ***p < 0.0001, 0.0008 (from left to right), two-way repeated-measures ANOVA.

To determine if the altered microbiome in *Dok3*^*−/−*^ mice is responsible for promoting enhanced colonic inflammation, we stimulated cells isolated from the colon lamina propria (LP) of WT mice with cecal contents from either WT or *Dok3*^*−/−*^ mice to mimic the cell-bacteria interactions in vivo during DSS-induced colitis. Interestingly, treatment of WT LP with cecal contents from *Dok3*^*−/−*^ mice triggered an increased production of pro-inflammatory cytokines associated with pathogenesis of IBD such as IL-23, TNFα, and IL-1β [19–22], as compared to treatment with cecal contents from WT mice (Fig. 3A), suggesting that the dysbiotic microbiome from *Dok3*^*−/−*^ mice is sufficient to drive colonic inflammation.

To demonstrate that the dysbiotic microbiome of *Dok3*^*−/−*^ mice contributes to their colitis susceptibility, we co-housed WT and *Dok3*^*−/−*^ mice to allow for the exchange of microbiota through coprophagia. Strikingly, co-housed *Dok3*^*−/−*^ mice exhibited diminished weight loss as compared to their singly-housed counterparts (Fig. 3B). This suggests that the enhanced susceptibility to colitis in *Dok3*^*−/−*^ mice can be reversed by the transfer of normal microbiota in a co-housing setting. We further performed 16S rRNA sequencing of fecal bacteria genomic DNA isolated from singly-housed or co-housed WT and *Dok3*^*−/−*^ mice to investigate the changes in microbiome upon co-housing. Based on the UniFrac distance matrix, the microbiome difference between singly-housed WT and *Dok3*^*−/−*^ mice was significantly greater than that between co-housed WT and *Dok3*^*−/−*^ mice, while the microbiome difference between co-housed and singly-housed *Dok3*^*−/−*^ mice was similar to that of singly-housed WT vs. *Dok3*^*−/−*^ mice (Fig. 3C). These data indicate that the community composition of microbiota in co-housed *Dok3*^*−/−*^ mice was closer in structure to that of WT mice. Specifically, the genera flavobacterium and rhodothermus were significantly reduced, while prevotella was significantly increased in co-housed *Dok3*^*−/−*^ mice as compared to singly-housed *Dok3*^*−/−*^ mice (Fig. 3D). Hence, the transferred microbiota from WT mice contributed to the ameliorated disease susceptibility of *Dok3*^*−/−*^ mice in a co-housing setting.

To allow for greater robustness in the normalization of microbiota, we also generated F2 *Dok3*^*+/+*^ and *Dok3*^*−/−*^ littermates from heterozygous crosses of mice [23]. In agreement with the co-housing study, *Dok3*^*+/+*^ and *Dok3*^*−/−*^ littermates which harbor similar intestinal flora show comparable colitis susceptibility, which was less severe than that seen in *Dok3*^*−/−*^ mice (Fig. 3E). Taken together, our findings demonstrate that both horizontal (via co-housing) and vertical (via breeding of littermates) transfer of microbiota from WT to *Dok3*^*−/−*^ mice protects *Dok3*^*−/−*^ mice against colitis development, and that the exacerbated colitis in *Dok3*^*−/−*^ mice is primarily driven by the lack of a protective microbiome.

### Enhanced production of S100a8/9 by colonic *Dok3*^*−/−*^ neutrophils

Recently, other members of the DOK family of proteins, namely DOK1 and DOK2, have been shown to regulate colitis severity through inhibiting the expression of Th17 cytokines IL17A and IL22 in DOK1/2 double knockout mice [24]. Hence, we speculate that DOK3 may function in a similar manner to control colitis susceptibility. However, according to our RT-qPCR analysis, *il17a* and *il22* expression levels were not significantly different between WT and *Dok3*^*−/−*^ LPs upon exposure to cecal contents, suggesting that DOK3 is likely to regulate colonic inflammation through a mechanism distinct from DOK1 and DOK2 (Supplementary Fig. 2).

**Fig. 4:**
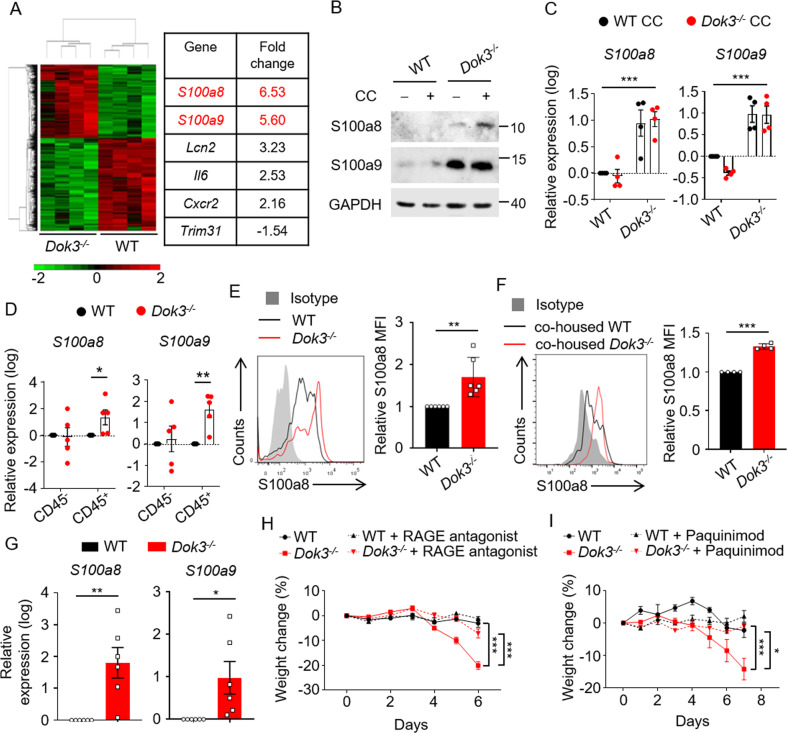
DOK3 regulates S100a8/9 production by neutrophils. **A** Expression heatmap based on microarray analysis of genes differentially expressed between colonic cells from WT and *Dok3*^*−/−*^ mice given 2% DSS in drinking water for 6 days. **B** Immunoblot analysis of S100a8, S100a9, and GAPDH in untreated (−) or cecal content (CC)-treated (+) lamina propria cells from WT and *Dok3*^*−/−*^ mice. Data is representative of three independent experiments. **C** RT-qPCR analysis of *S100a8* and *S100a9* expression relative to *b-actin* expression in lamina propria cells of WT and *Dok3*^*−/−*^ mice following 3 h stimulation with cecal contents from WT and *Dok3*^*−/−*^ mice. Data is shown as mean±S.E.M (n = 4, four independent experiments). ***p < 0.0001, two-way ANOVA. **D** RT-qPCR analysis of *S100a8* and *S100a9* expression relative to *b-actin* expression in CD45^−^ and CD45^+^ cell fractions isolated from the lamina propria of WT and *Dok3*^*−/−*^ mice following 3 h stimulation with cecal contents from WT mice. Data is shown as mean ± SEM (*n* = 5, five independent experiments). *p = 0.04, **p = 0.002, unpaired two-tailed Student’s *t*-test. **E** Flow cytometric analysis of S100a8 expression in WT and *Dok3*^*−/−*^ neutrophils following 3 h stimulation with cecal contents from WT mice. Histograms were pre-gated on singlet, CD45^+^, Ly6G^+^ cells. Filled histogram represent isotype control. Bar graph depicting MFI of S100a8 fluorescence in *Dok3*^*−/−*^ neutrophils relative to WT neutrophils. Data is shown as mean ± SD (*n* = 6, five independent experiments). **p = 0.004, unpaired two-tailed Student’s *t*-test. **F** Flow cytometric analysis of S100a8 expression in co-housed WT and *Dok3*^*−/−*^ neutrophils following 3 h stimulation with cecal contents from WT mice. Histograms were pre-gated on singlet, CD45^+^, Ly6G^+^ cells. Filled histogram represent isotype control. Bar graph depicting MFI of S100a8 fluorescence in *Dok3*^*−/−*^ neutrophils relative to WT neutrophils. Data is shown as mean ± SD (n = 4, three independent experiments). **p < 0.0001, unpaired two-tailed Student’s *t*-test. **G** RT-qPCR analysis of *S100a8* and *S100a9* expression relative to *b-actin* expression in bone marrow-purified neutrophils from WT and *Dok3*^*−/−*^ mice following 3 h stimulation with cecal contents from WT mice. Data is shown as mean±S.E.M (*n* = 6, six independent experiments). *p = 0.02, **p = 0.003, unpaired two-tailed Student’s *t*-test. **H** WT and *Dok3*^*−/−*^ mice were injected intraperitoneally with RAGE antagonist (1 mg/kg) daily, and given 2% DSS in their drinking water for 7 days. Body weights were measured daily. Data is shown as mean ± SEM (*n* = 4). ***p < 0.0001, two-way repeated-measures ANOVA. **I** WT and *Dok3*^*−/−*^ mice were injected intraperitoneally with Paquinimod (5 mg/kg) daily, and given 2% DSS in their drinking water for 8 days. Body weights were measured daily. Data is shown as mean ± SEM (*n* = 4). *p = 0.03, ***p < 0.0001, two-way repeated-measures ANOVA.

To understand what underlying mechanism results in the altered microbiota and hence exacerbated colitis in *Dok3*^*−/−*^ mice, we performed microarray-based transcriptome analysis of DSS-treated WT and *Dok3*^*−/−*^ colons to identify genes which were differentially expressed (DEGs) between WT and *Dok3*^*−/−*^ colonic cells during colitis. 916 DEGs were identified in total, of which 439 were upregulated and 477 were down-regulated in *Dok3*^*−/−*^ compared with WT colonic cells (restricted to those with fold change >1.5; unpaired Student’s *t*-test, *p* < 0.05). From the list, we highlighted two highly upregulated genes-of-interest, *S100a8/a9* (Fig. 4A), which have been widely implicated as inflammatory mediators of IBD [25, 26], and can perturb the growth of bacterial communities [27–31]. The increased expression of *S100a8/a9* in *Dok3*^*−/−*^ colons during DSS-induced colitis was further validated by reverse transcription-quantitative polymerase chain reaction (RT-qPCR) analysis (Supplementary Fig. 3). Moreover, S100a8/9 were also increased at the protein level in *Dok3*^*−/−*^ LP as compared to WT LP (Fig. 4B). Since microbiome can affect colonic gene expression as shown earlier (Fig. 3A), we investigated whether higher levels of *S100a8/a9* were caused by a dysbiotic microbiome in the colons of *Dok3*^*−/−*^ mice. We exposed WT and *Dok3*^*−/−*^ LP to cecal contents from WT and *Dok3*^*−/−*^ mice, and observed elevated levels of *S100a8/a9* in the *Dok3*^*−/−*^ LP as compared to WT LP, regardless of stimulation with cecal contents from WT or *Dok3*^*−/−*^ mice (Fig. 4C). This shows that the enhanced production of S100a8/9 is a direct result of the genetic deficiency in *Dok3*.

S100a8/9 proteins are primarily expressed by neutrophils and epithelial cells during inflammation [26]. Hence, we sought to determine which cellular compartment(s) in the colon LP of *Dok3*^*−/−*^ mice contributes to the elevated expression of *S100a8/a9*. We first purified CD45^+^ and CD45^−^ compartments from WT and *Dok3*^*−/−*^ LP, and stimulated them with cecal contents from WT mice to minimize variability since colonic LP produces similar amounts of *S100a8/a9* in response to cecal contents from either WT or *Dok3*^*−/−*^ mice (Fig. 4C). Stimulation with WT cecal content significantly increased the expression of *S100a8/a9* in CD45^+^ but not CD45^−^ cells from *Dok3*^*−/−*^ compared with WT mice (Fig. 4D). This suggests that immune cells in *Dok3*^*−/−*^ colon contributed to S100a8/9 hyper-production, and hence microbial dysbiosis and enhanced inflammation, during colitis. To further characterize these immune cells, we stained for S100a8 expression in colonic neutrophils, macrophages and DCs by flow cytometry (Supplementary Fig. 4A). We focused on innate immune instead of adaptive cells since S100a8/9 proteins are expressed predominantly by innate cells [26], and DSS-induced colitis is a model for the study of innate immune mechanisms [32]. Interestingly, we saw an increased expression of S100a8 in *Dok3*^*−/−*^ compared with WT neutrophils upon stimulation of LP with cecal contents (Fig. 4E). To eliminate the possibility that enhanced S100a8 production by neutrophils is a bystander effect due to altered colonic microbiota in *Dok3*^*−/−*^ mice, we further stained for S100a8 expression in colonic neutrophils isolated from co-housed WT and *Dok3*^*−/−*^ mice, which harbor similar intestinal flora (Fig. 3C, D). We observed that S100a8 level remains elevated in co-housed *Dok3*^*−/−*^ colonic neutrophils, suggesting that the difference in S100a8 production is not triggered by the dysbiotic microbiome (Fig. 4F). In line with this, when we purified bone marrow neutrophils from WT and *Dok3*^*−/−*^ mice and stimulated them in vitro with cecal contents, higher levels of *S100a8/9* transcripts were observed in *Dok3*^*−/−*^ bone marrow neutrophils, indicating an intrinsic defect in their ability to suppress *S100a8/9* production (Fig. 4G). Moreover, S100a8 expression was not significantly different in colonic macrophages, DCs, and other non-immune cells from WT and *Dok3*^*−/−*^ mice (Supplementary Fig. 4B). Thus, these results demonstrate that DOK3 suppresses the expression of inflammatory proteins S100a8/9 in neutrophils, thereby maintaining gut microbial ecology and colon homeostasis.

To demonstrate the causal relationship between S100a8/9 levels and the exacerbated colitis phenotype in *Dok3*^*−/−*^ mice, we treated WT and *Dok3*^*−/−*^ mice with the RAGE antagonist FPS-ZM1, which can inhibit the function of S100a8/9 by blocking their binding to the RAGE receptor [33]. We observed that delivery of RAGE antagonist is able to attenuate the colitis susceptibility of *Dok3*^*−/−*^ mice (Fig. 4H). Similarly, treatment with Paquinimod, an inhibitor of S100a9, can also rescue colitis severity of *Dok3*^*−/−*^ mice (Fig. 4I). Together, our findings indicate that elevated levels of S100a8/9 contributed to the enhanced colonic inflammation in *Dok3*^*−/−*^ mice.

### DOK3 suppresses S100a8/9 expression by limiting JAK2-STAT3 signaling in neutrophils

**Fig. 5:**
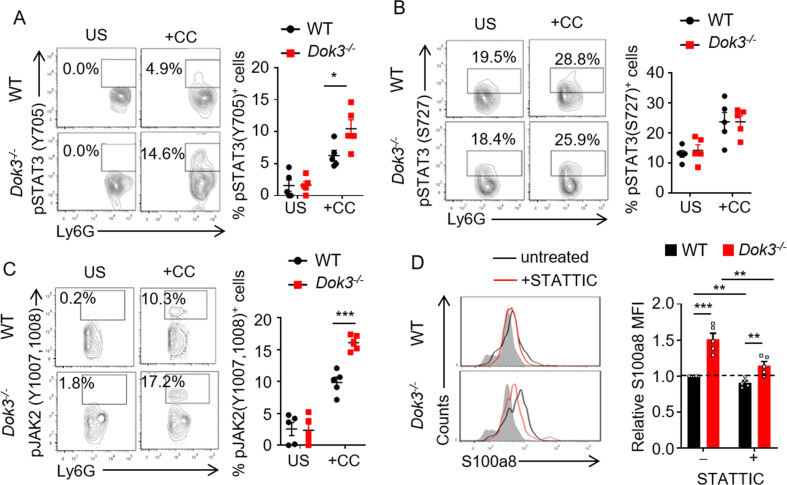
DOK3 suppresses JAK2-STAT3 signaling in neutrophils. **A**–**C** Lamina propria cells from WT and *Dok3*^*−/−*^ mice were unstimulated (US) or stimulated for 10 min with cecal contents (+CC) from wild-type mice. Flow cytometric analysis of **A** pSTAT3 (Y705), **B** pSTAT3 (S727) and **C** pJAK2 (Y1007, 1008) in colonic neutrophils (*n* = 5, four independent experiments). Contour plots were pre-gated on singlet, CD45^+^, Ly6G^+^ cells. Data is shown as mean ± SEM. **A** *p = 0.03, unpaired two-tailed Student’s *t*-test. **C** ***p = 0.0002, unpaired two-tailed Student’s *t*-test. **D** Cells from lamina propria of WT and *Dok3*^*−/−*^ mice were stimulated for 3 h with cecal contents from wild-type mice in the absence (black histogram) or presence (red histogram) of STATTIC. Flow cytometric analysis of S100a8 expression in WT and *Dok3*^*−/−*^ colonic neutrophils. Histograms were pre-gated on singlet, CD45^+^, Ly6G^+^ cells. Filled histogram represents isotype control. Bar graph depicts MFI of S100a8 fluorescence in WT (black bars) and *Dok3*^*−/−*^ (red bars) neutrophils treated or not with STATTIC relative to that of untreated WT neutrophils. Data is shown as mean ± SEM (*n* = 5, four independent experiments). **p = 0.008, 0.007, 0.005 (from left to right), ***p = 0.0002, unpaired two-tailed Student’s *t*-test.

We next explored the signaling pathways involved in DOK3-mediated suppression of S100a8/9 in neutrophils. S100a8/9 production has been shown to be regulated via a STAT3-dependent pathway [34, 35]. Here, we observed that stimulation of WT colons with cecal contents induced minimal phosphorylation of STAT3 at Tyr705 (which is indicative of STAT3 activation) in neutrophils (Fig. 5A), consistent with the notion that LP innate cells are hypo-responsive to bacterial stimulation to maintain tolerance in the intestine [36]. In contrast, cecal contents significantly increased the levels of phosphorylated STAT3 at Tyr705 in *Dok3*^*−/−*^ neutrophils, suggesting that DOK3 is required to maintain colonic neutrophils in a quiescent state (Fig. 5A). We further analyzed but observed no difference in the levels of STAT3 phosphorylation on Ser727 (a secondary phosphorylation site for the inactivation of STAT3) between WT and *Dok3*^*−/−*^ neutrophils (Fig. 5B). Moreover, comparable expression of S100a8 in WT and *Dok3*^*−/−*^ colon LP cell types apart from neutrophils (CD45^-^ and CD45^+^ Ly6G^−^ cells) was congruent with similar STAT3 phosphorylation at both Tyr705 and Ser727 observed in these cells (Supplementary Fig. 5). Since STAT3 is phosphorylated by JAKs at Tyr705 [37], we investigated if DOK3 deficiency affects JAK2 activation. Indeed, stimulation with cecal contents enhanced the phosphorylation and hence, activation of JAK2 in *Dok3*^*−/−*^ neutrophils (Fig. 5C). Subsequently, to validate the direct involvement of JAK2/STAT3 pathway in promoting S100a8 expression, we treated WT and *Dok3*^*−/−*^ colons with a selective STAT3 inhibitor, STATTIC [38]. Inhibition of STAT3 is able to reduce S100a8 expression, and the effect is more pronounced with *Dok3*^*−/−*^ than WT neutrophils (Fig. 5D). Together, these data show that DOK3 restrains JAK2-STAT3 pathway to suppress S100a8/9 production in colonic neutrophils upon exposure to cecal contents, thereby regulating gut microbial community and maintaining homeostasis in the colon.

## Discussion

The possibility that DOK3 might play a role in IBD was first revealed in a GWAS study of Crohn’s disease and ulcerative colitis [1], but experimental evidence definitively establishing their causal link was lacking. In this study, we demonstrated that DOK3 plays a protective role in experimental colitis by restraining JAK2/STAT3 signaling in colonic neutrophils in response to commensal microbes. In this way, it preserves quiescence in colonic neutrophils and suppresses the excessive production of S100a8/9 and thereby limits intestinal inflammation to ensure a balanced gut microbiome. In the intestinal microenvironment, where immune cells are constantly being challenged by commensal microbes and dietary antigens, maintaining tolerance is essential to prevent detrimental immune responses leading to diseases such as IBD.

Pathogenesis of IBD has been known to depend critically on host genetic factors as well as gut microbiome, but our knowledge on how the immune system can regulate growth and composition of the microbial communities remains limited. Here, we demonstrated that DOK3 can regulate the gut microbiota by limiting the spontaneous release of S100a8/9 by colonic neutrophils in response to cecal contents. S100a8/9 are able to constrain commensal bacterial growth and disrupt bacterial communities by sequestration and chelation of essential metal nutrients such as manganese and zinc in tissues [27, 29–31], thus rendering bacteria more sensitive to neutrophil-mediated killing. In addition, they can enhance NADPH oxidase activity and promote autophagy to inhibit growth of bacteria [28]. As a result, changes in gut microbiota composition, in particular reduced abundance of Bacteriodes and Prevotella, was observed in patients with increased fecal levels of S100a8/9. In our study, excessive production of S100a8/9 by neutrophils in *Dok3*^*−/−*^ mice consequently promoted microbiome dysbiosis in their gut, resulting in mutant mice succumbing to exacerbated colitis upon DSS treatment. In line with their greater sensitivity to DSS-induced colitis, we saw a loss of protective intestinal bacterial genera such as Bacteriodes in untreated *Dok3*^*−/−*^ mice, which correlates with what was observed in the microbiota obtained from IBD patients with elevated fecal levels of S100a8/9 [16–18]. Concurrently, Flavobacterium was more highly enriched in the fecal microbiota of *Dok3*^*−/−*^ mice. Such bacteria has been shown to induce high levels of pro-inflammatory cytokines in various organisms upon infection [39–41]. Indeed, cecal contents from *Dok3*^*−/−*^ mice enriched with Flavobacterium were able to trigger greater production of pro-inflammatory cytokines by WT LP. In addition, *Dok3*^*−/−*^ mice exhibited higher levels of colonic inflammation upon DSS treatment, as evidenced by the increased activation of NF-κB and STAT3 and greater infiltration of immune cells into the gut. Consequently, upon blockage of S100a8/9 binding to RAGE or TLR4 receptor through administration of FPS-ZM1 or Paquinimod, we were able to rescue colitis severity in *Dok3*^*−/−*^ mice, indicating the importance of S100a8/9 in promoting colonic inflammation. However, we cannot exclude the possible contribution of RAGE or TLR4 receptor on colitis in *Dok3*^*−/−*^ mice, and more work needs to be done in future to address this. Taken together, loss of DOK3 initially drives the release of pro-inflammatory mediators S100a8/9 by colonic neutrophils upon exposure to bacteria, and this perturbs the balance of gut microbiome and promotes the outgrowth of colitogenic strains, which in turn triggers further inflammation in the gut, ultimately escalating the severity of colitis.

Gain-of-function variants of *JAK2* and *STAT3* have been reported to be associated with IBD progression, and various small-molecule inhibitors of JAK have been shown to be efficacious in the treatment of IBD over the past years, especially for patients who are refractory to treatment with TNFα antagonists [42, 43]. This therapeutic approach is based on the rationale that JAK-STAT mediated signaling regulates multiple cytokine-dependent pathways involved in the pathogenesis of IBD, including IL-6, IL-10, and IL-23 [44], and such inhibitors are able to target multiple immune pathways simultaneously to improve response in patients. However, it is increasingly evident that although active IBD is associated with hyper-activation of JAKs, the expression and regulation of JAKs within each gut cell type is different, and depending on the ligand-receptor pair, activation of JAK can lead to a multitude of distinct outcomes. Thus, it is important to have a deeper understanding of JAK-mediated signaling pathways for optimization of therapies employing such small-molecule JAK inhibitors to ensure their clinical safety and efficacy. In this study, we report novel findings on how excessive JAK-STAT signaling in DOK3-deficient neutrophils drives the production of S100a8/9, thereby disrupting the gut microbial ecology, ultimately leading to intestinal inflammation. This pathway exists exclusively in neutrophils, as we do not observe similar dysregulation of JAK2-STAT3 signaling and S100a8/9 production in other *Dok3*^*−/−*^ colonic cell types, further reinforcing the differential contribution of JAKs in different cell types to intestinal homeostasis. Indeed, JAK2 expression was previously found to be restricted to the myeloid compartment in the human intestine mucosa [42]. Consistent with our previous study showing DOK3 to be highly expressed in neutrophils compared with other immune cell types [11], it is likely that specific expression of JAK2 and DOK3 plays a role in defining their intrinsic function in neutrophils. Our findings therefore further exemplify the complexity and cell type-dependence of JAK-STAT signaling in the gut during intestinal homeostasis and inflammation. However, future work needs to be done to address how adapter protein DOK3 can negatively regulate JAK2 activity, which is likely to be mediated through other interacting enzymatic protein partner(s).

In summary, our study demonstrated that DOK3 suppresses JAK2-STAT3 signaling to limit S100a8/9 production and imprint a non-inflammatory state in colonic neutrophils in response to gut bacteria, thereby playing an essential role in promoting protective commensal communities that protect against intestinal inflammation. Such host microbial cross-talk is critical for the maintenance of intestinal homeostasis. Since S100a8/9 expression has been shown to correlate with clinical severity in IBD patients, and their neutralization can attenuate colitis pathology, targeting JAK2-STAT3 signaling axis may represent potential therapeutic strategies to regulate gut microbiome and inflammation.

## Materials and methods

### Mice

C57BL/6 mice were purchased from The Jackson Laboratory. *Dok3*^*−/−*^ mice were generated as described previously [45]. *Dok3*^*−/−*^ mice were backcrossed to C57BL/6 mice for more than ten generations. Male and female mice were used at 8–10 weeks of age unless otherwise stated. All mice were maintained under specific pathogen-free conditions at A*STAR Biological Resource Center (BRC).

### Experimental colitis

Mice were treated with 2% (w/v) DSS (Sigma-Aldrich) dissolved in sterile, distilled water ad libitum for 7 days followed by normal drinking water till end of experiment. Body weights and mouse survival were monitored daily. To block the effects of S100a8/9, mice were injected intraperitoneally with 5 mg/kg of Paquinimod or 1 mg/kg of FPS-ZM1 daily.

### Histology

Colons were swiss rolled and fixed in 10% neutral-buffered formalin before embedding in paraffin wax. Colon sections were stained with hematoxylin and eosin (H&E), Ki67, neutrophil elastase, and CD11b.

### Flow cytometry

Cell suspensions were surface labeled with fluorochrome-conjugated antibodies for 10 m at 4 °C in staining buffer (PBS containing 1% BSA). For phosphoprotein staining, cells were fixed and permeabilized using the Phosflow kit (BD) according to manufacturer’s protocol before staining for 1 h at room temperature. Data were acquired using LSRII (BD Biosciences) and analyzed using FlowJo software (Tree Star). The following antibodies were used for flow cytometry analysis: anti-CD45 APC/Cy7 (clone 30-F11; BioLegend), anti-Ly6G PE/ biotin (clone 1A8; BD, BioLegend), anti-CD11c PerCP (clone N418; BioLegend), anti-F4/80 PE (clone BM8; eBioscience), anti-I-A/I-E FITC (clone M5/114.152; BioLegend), anti-Gr1 (clone RB6–8C5; BD Pharmingen), anti-CD11b (clone M1/70; BD Pharmingen), anti-STAT3 (Y705) PE (clone 4/P-STAT3, BD Biosciences), anti-STAT3 (S727) PE (clone 49/P-STAT3, BD Biosciences), anti-pJAK2 (Y1007,1008) Alexa Fluor 647 (clone E132, abcam), anti-S100a8 (catalog PA5-86063, ThermoFisher Scientific).

### Western blotting

Colonic cells were lysed with cell lysis buffer (Cell Signaling Technology) containing protease and phosphatase inhibitors (Cell Signaling Technology). Cell lysates were analyzed by Western blotting according to standard protocol using the indicated antibodies: anti-pSTAT3 (Y705) (clone D3A7; Cell Signaling Technology), anti-STAT3 (clone 79D7; Cell Signaling Technology), anti-pp65 (clone 93H1; Cell Signaling Technology), anti-p65 (clone D14E12; Cell Signaling Technology), anti-S100a8 (clone E4F8V; Cell Signaling Technology), anti-S100a9 (clone D3U8M; Cell Signaling Technology).

### Isolation of mouse LP

Colons were opened longitudinally and fecal contents were removed by washing with PBS. Colons were cut into 1 cm pieces and predigested in HBSS containing EDTA and DTT with shaking at 37 °C for 30 m. After washing, colons were finely minced and digested in PBS containing collagenase D (Roche), dispase (Roche) and DNase I (Sigma) with shaking at 37 °C for 30 m. After digestion, cell suspensions were filtered through a cell strainer to obtain the LP cells.

### Isolation of neutrophils

Neutrophils were isolated from tibias and femurs of mice using anti-Ly-6G magnetic beads (Miltenyi Biotec). Purity of isolated cells was confirmed by flow cytometry.

### Microarray analysis

Colonic cells were isolated from WT and *Dok3*^*−/−*^ mice before and 6 days after DSS challenge (*n* = 4 each). Total RNA was extracted using TRIzol (Invitrogen) and precipitated with isopropanol, followed by DNase I digestion using RNase-Free DNase Set and purification using RNeasy MinElute Cleanup Kit (both kits from Qiagen). After quantification with NanoDrop ND-2000 spectrophotometer and determination that the RNA integrity number (RIN) of all RNA samples were >8.5 with Agilent 2100 Bioanalyzer, single-stranded cDNA was then prepared and hybridized on a GeneChip Mouse Gene 2.0 ST Array (Affymetrix). Significantly differentially expressed genes (DEGs) between *Dok3*^*−/−*^ and WT colonic cells before and after DSS challenge (fold change >1.5-fold; two-tailed *t*-test with equal variances, *p* < 0.05) were detected using Partek Genomics Suite (PGS) software (Partek Inc.). GSEA was performed to identify the most significantly enriched gene sets corresponding to specific cellular and transcriptional pathways based on DEGs between *Dok3*^*−/−*^ and WT cells.

### Stool gDNA extraction and microbiome analysis

Fecal pellets were freshly removed from colons of mice and gDNA was extracted using QIAamp Fast DNA Stool Mini Kit (Qiagen), according to manufacturer’s protocol. Libraries were prepared by amplifying 16S rRNA V3−V4 gene region and sequenced on the Illumina MiSeq platform to generate paired-end 2 × 250 bp reads (AITbiotech). The MiSeq Reporter Metagenomics workflow was performed to conduct taxonomic classification of the reads according to the Greengenes Database v13.5 (http://greengenes.lbl.gov/) and generate read counts of each taxon present in the gut microbiome. The raw read counts were converted to relative proportions per sample and normalized to shifted logit-p proportions as described previously [46]. The normalized proportions were used to identify taxa of significantly different abundance using two-tailed *t*-test with unequal variance *p* < 0.05. Beta-diversity analysis (weighted and unweighted UniFrac distances) was conducted using QIIME2. Kruskal–Wallis tests with Dunn’s post-hoc tests were used to evaluate the effects of co-housing on beta-diversity.

### Quantitative PCR

Purified neutrophils were lysed with TRIzol (Gibco, Thermo Fisher Scientific), and RNA was purified using phenol/chloroform extraction. Complementary DNA was reversed transcribed using RevertAid First Strand cDNA Synthesis Kit (Thermo Fisher Scientific). The following primers were used for real-time PCR using SYBR Green PCR Master Mix (Applied Biosystems): 16S rDNA (forward): AGAGTTTGATCMTGGCTCAG; 16S rDNA (reverse): CTGCTGCCTYCCGTA; 18S rDNA (forward): ATTGGAGGGCAAGTCTGGTG; 18S rDNA (reverse): CCGATCCCTAGTCGGCATAG; S100a8 (forward): TGTCCTCAGTTTGTGCAGAATATAAA; S100a8 (reverse): TCACCATCGCAAGGAACTCC; S100a9 (forward): GGTGGAAGCACAGTTGGCA; S100a9 (reverse): GTGTCCAGGTCCTCCATGATG; IL6 (forward): GAGGATACCACTCCCAACAGACC; IL6 (reverse): AAGTGCATCATCGTTGTTCATACA; IL23 (forward): GCAGATTCCAAGCCTCAGTC; IL23 (reverse): TTCAACATATGCAGGTCCCA; IL1β (forward): CAACCAACAAGTGATATTCTCCATG; IL1β (reverse): GATCCACACTCTCCAGCTGCA; TNFα (forward): GCCTCTTCTCATTCCTGCTTG; TNFα (reverse): CTGATGAGAGGGAGGCCATT; β-actin (forward): AGATGACCCAGATCATGTTTGAGA; β-actin (reverse): CACAGCCTGGATGGCTACGTA.

### Statistics

Figures and statistical analyses were generated using Graphpad Prism software. Mice were allocated to experimental groups based on genotypes and were randomized within their sex-matched and age-matched groups. No mouse was excluded from the analyses. For weight loss analyses, two-way repeated-measures ANOVA was performed. For survival analyses, log-rank test was performed. For other analyses, unpaired two-tailed Student’s *t*-test was performed. A *p* value of less than 0.05 was considered significant.

## Supplementary information

Supplementary figures

## Data Availability

The microarray data has been deposited into NCBI GEO, accession number GSE186597.
