# Supplementary material for: DOK3 maintains intestinal homeostasis by suppressing JAK2/STAT3 signaling and S100a8/9 production in neutrophils
Source: Cell Death Dis. 2021 Nov 6;12(11):1054. doi: 10.1038/s41419-021-04357-5 (PMC8572282; doi:10.1038/s41419-021-04357-5)
Supplement: Supplementary file 1 — Supplementary figures [file 41419_2021_4357_MOESM1_ESM.docx]

**Supplementary Figures**

**
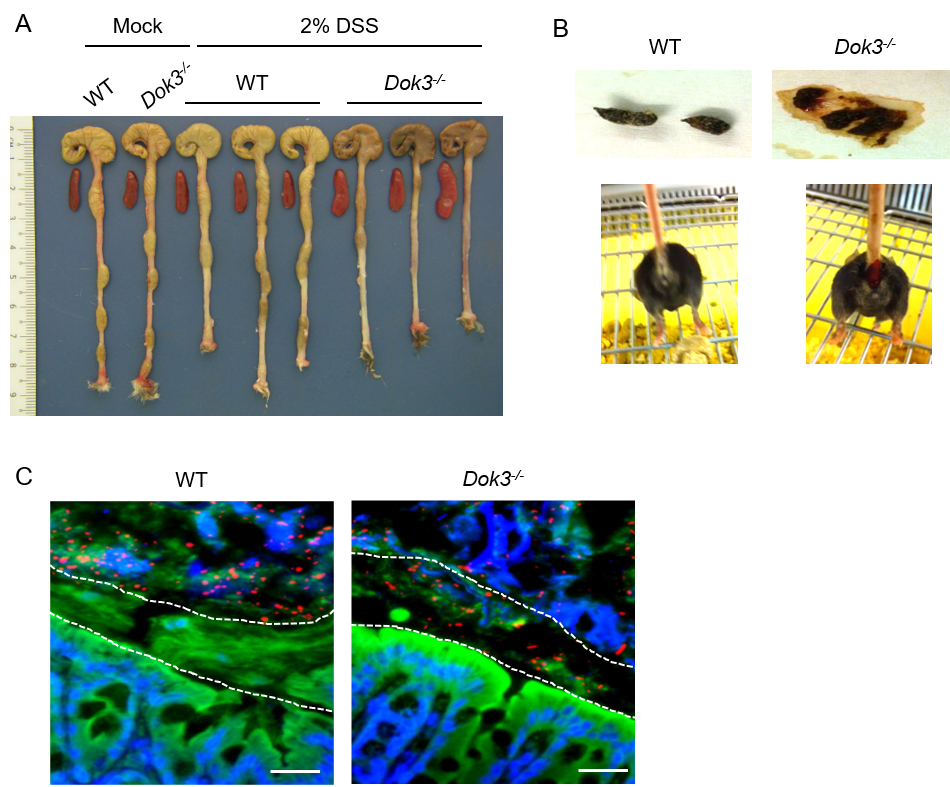
**

**Supplementary Figure 1. Loss of DOK3 exacerbates DSS-induced colitis.** **A** WT and *Dok3^-/-^* mice were given 2% DSS *ab libitum* in their drinking water for 7 days. Colons and spleens were harvested on day 8 after initiation of DSS treatment. **B** Representative photographs of feces (top) and rectal bleeding (bottom) of WT and *Dok3^-/-^* mice following DSS treatment. **C** Destruction of mucin layer (green) and bacterial (red) penetration in colons of *Dok3^-/-^* mice following DSS treatment. Dotted lines demarcate the mucin layer. Magnification 40x.

**
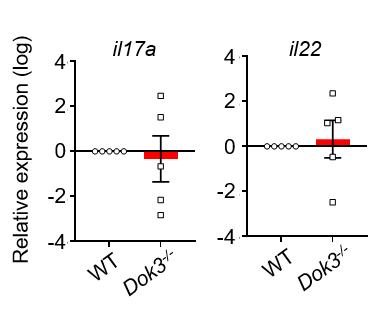
**

**Supplementary Figure 2. DOK3 does not regulate IL17A and IL22 expression.** RT-qPCR analysis of *Il17a* and *il22* expression relative to *b-actin* expression in WT and *Dok3^-/-^* LP following 3h stimulation with cecal contents. Data is shown as mean±S.E.M (n=5, 5 independent experiments).

**
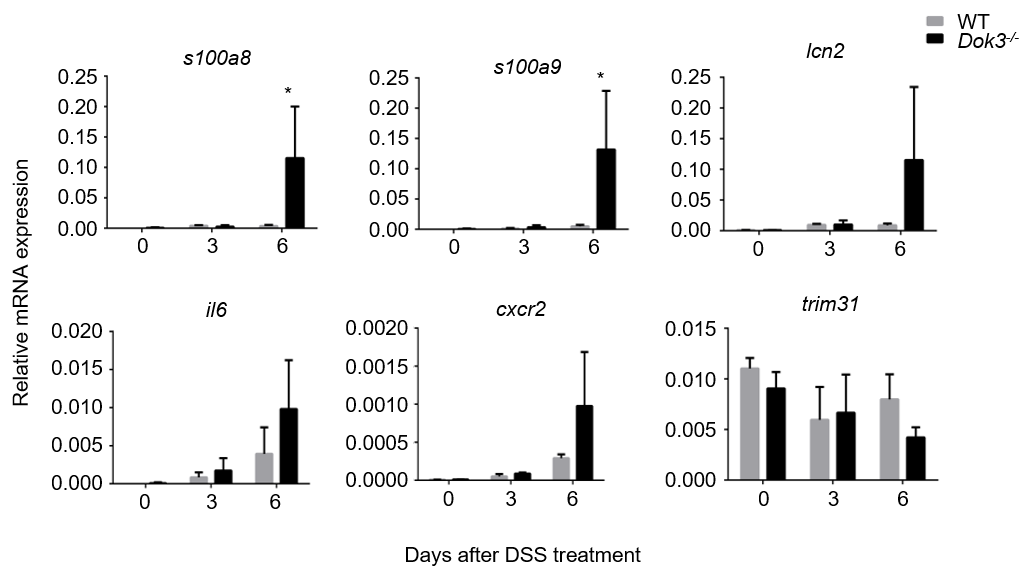
**

**Supplementary Figure 3. Validation of differentially expressed genes in the colons of WT and *Dok3^-/-^* mice.** Mice were treated with 2% DSS in drinking water, and colons were harvested on day 0, 3, and 6 after initiation of DSS treatment. RT-qPCR analysis of *S100a8*, *S100a9*, *lcn2*, *il6*, *cxcr2* and *trim31* expression relative to *36B4* expression in the colons of WT and *Dok3^-/-^* mice (n=3). *p=0.04, 0.04 (from left to right), unpaired two-tailed Student’s t-test.

**
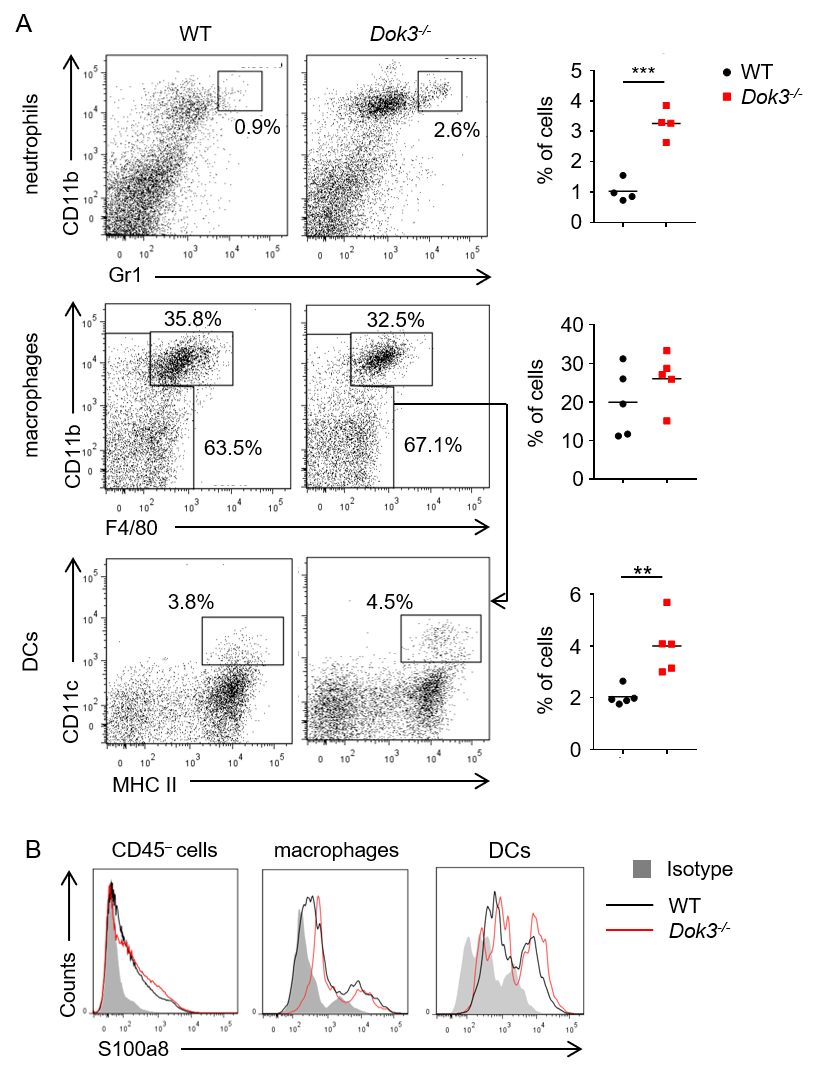
**

**Supplementary Figure 4. Expression of S100a8 in various cell types in the colons.** **A** Gating strategy for neutrophils (top), macrophages (middle) and dendritic cells (DCs) (bottom) using flow cytometry. Dot plots were pre-gated on singlet, CD45^+^ cells. Graph (right) indicates percentage of neutrophils, macrophages and DCs in the lamina propria of WT and *Dok3^-/-^* mice (n=4-5, 4-5 independent experiments). **p=0.005, ***p=0.0004, unpaired two-tailed Student’s t-test. **B** Flow cytometric analysis of S100a8 expression in WT and *Dok3^-/-^* CD45^-^ cells, macrophages and DCs. Histograms were pre-gated as indicated in **A**. Filled histogram represent isotype control.

**
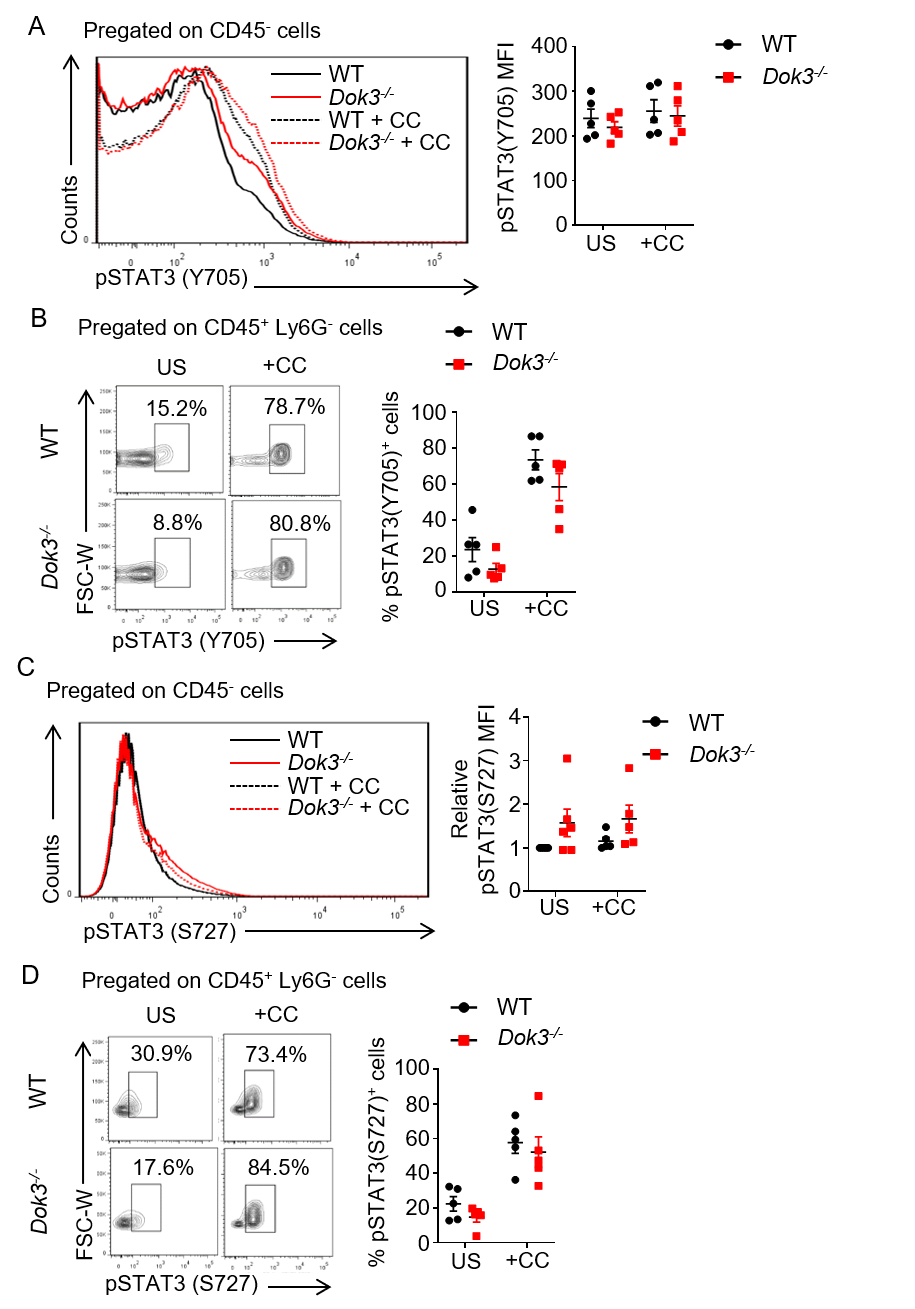
**

**Supplementary Figure 5. STAT3 signaling in various cell types in the colons. A-D** Lamina propria from WT and *Dok3^-/-^* mice were unstimulated (US) or stimulated for 10 min with cecal contents (+CC). Flow cytometric analysis of **A,B** pSTAT3 (Y705) and **C,D** pSTAT3 (S727) on **A,C** CD45^-^ cells or **B,D** CD45^+^ Ly6G^-^ cells in the lamina propria. **A,C** Histograms were pre-gated on singlet, CD45^-^ cells. **B,D** Contour plots were pre-gated on singlet, CD45^+^, Ly6G^-^ cells. Data is shown as mean±S.E.M (n=5, 4 independent experiments).
